# Nonwoven Releasing Propolis as a Potential New Wound Healing Method—A Review

**DOI:** 10.3390/molecules26185701

**Published:** 2021-09-21

**Authors:** Mateusz Stojko, Daniel Wolny, Jakub Włodarczyk

**Affiliations:** 1 Center of Polymer and Carbon Materials, Polish Academy of Sciences, Marii Curie-Skłodowskiej 34, 41-819 Zabrze, Poland; jwlodarczyk@cmpw-pan.edu.pl; 2 Department of Biopharmacy, Faculty of Pharmaceutical Sciences in Sosnowiec, Medical University of Silesia in Katowice, Jedności 8, 41-200 Sosnowiec, Poland; dwolny@sum.edu.pl

**Keywords:** propolis, wound, dressing, biodegradable, polymer, nonwoven

## Abstract

Wound healing poses a serious therapeutic problem. Methods which accelerate tissue regeneration and minimize or eliminate complications are constantly being sought. This paper is aimed at evaluation of the potential use of biodegradable polymer nonwovens releasing propolis as wound healing dressings, based on the literature data. Propolis is honeybee product with antioxidant, antibacterial, antifungal, anticancer, anti-inflammatory, analgesic, and regenerative properties. Controlled release of this substance throughout the healing should promote healing process, reduce the risk of wound infection, and improve aesthetic effect. The use of biodegradable aliphatic polyesters and polyester carbonates as a propolis carrier eliminates the problem of local drug administration and dressing changes. Well-known degradation processes and kinetics of the active substance release allows the selection of the material composition appropriate to the therapy. The electrospinning method allows the production of nonwovens that protect the wound against mechanical damage. Moreover, this processing technique enables adjusting product properties by modifying the production parameters. It can be concluded that biodegradable polymer dressings, releasing a propolis, may find potential application in the treatment of complicated wounds, as they may increase the effectiveness of treatment, as well as improve the patient’s life quality.

## 1. Background

Specialists all over the world struggle with the therapeutic problem of wound healing, which reduces many patients’ life quality. The inconvenience of the healing processes of complex wounds means that modern methods of treatment, which will be able to accelerate tissue regeneration and eliminate complications related to this process, are constantly being sought [1].

Skin, which is the largest human organ, is the main barrier with immune, sensory, and protective functions, and it is exposed to various types of damage and injuries [1]. Due to the deteriorating health condition of the population, associated with the increasing prevalence of obesity and chronic diseases, such as circulatory failure and diabetes, the frequency of chronic wounds increases [2]. Chronic wounds are estimated to affect approximately 1–2% of the population in Europe and the United States [2]. Extensive burn wounds are also important therapeutic problem, which may pose a threat to the patient’s life [3]. People affected by the problem of difficult-to-heal wounds struggle not only with pain, excessive exudates, unpleasant smell of the wound, and limited mobility but also with their consequences, such as limitations of social life [2]. A large number of patients, expecting better effects of therapy, and significant funds allocated to their treatment, contribute to the development of research in the field of wound regeneration and treatment.

The global wound care market reached $19.8 billion in 2019 and is forecast to grow to $24.8 billion in 2024, resulting in a Compound Annual Growth Rate (CAGR) of 4.6%. The reasons of the increase are: increase in the number of road injuries and accidents, growing use of regenerative medicine, and increasing prevalence of diseases that impair the ability to heal wounds. In 2019, advanced wound care represented the largest part of this market, and this type of product is responsible for the largest part of the growth of this market value [4].

## 2. Wounds

A wound is damage to the anatomical continuity of a tissue caused by different factors. The skin has its own regenerative potential. Injuries heal as a result of a highly organized cascade of physiological processes [5].

In some cases, the regenerative properties are impaired, and the duration of repair processes is significantly prolonged, which exposes patients to health complications. Treatment of chronic wounds and extensive burns is expensive and time-consuming as they are prone to infection and often require surgical intervention. The treatment of this type of wound places a heavy burden on the health care system, and the risk of chronic wounds is constantly increasing [3,6,7].

The oldest methods of treating wounds consist of covering the wound and applying natural ointments to reduce pain, prevent infection, and protect the wound. Although a similar procedure is used today, it is insufficient in the case of chronic wounds. Much attention is now being paid to the development of modern methods of treatment that will allow the regeneration of damaged skin. Studies conducted in this field are related to:

- identification of processes involved in skin regeneration,
- disturbances in regenerative processes in chronic wounds,
- drug dosing systems enabling effective delivery of substances to the wound bed,
- development of materials that act as a scaffold for cell growth, and
- development of advanced dressings [3].

There are many different ways of classifying wounds, but the most commonly used are divisions according to the cause of their formation, healing time, and the depth of damage [8,9].

The division according to the cause of the occurrence includes: abrasions and scratches, cut wounds, stab wounds, bruised wounds, lacerations, flap wounds, scalping wounds, bitten wounds, poisoned wounds, and burn wounds [8,9,10,11]. Depending on the healing time, wounds can be divided into acute wounds (healing within 6 weeks from the wound formation) and chronic wounds (healing within more than 6 weeks from the wound formation) [9]. According to the depth of damage, wounds can be divided into:

- superficial-not exceeding the subcutaneous tissue; and
- deep-reaching beyond the subcutaneous tissue [9,10,11].

Burns are a special type of wounds, and they are classified separately according to the area and depth of the damage. The wound surface is important due to the large loss of fluid through the damaged epidermis [12].

Besides local damage to the skin and surrounding tissues, burns affect the entire body. They increase the permeability of capillaries, which leads to leakage of plasma from the vessels into the interstitial spaces, which reaches its maximum in the first 8 h and lasts up to 48 h. After this time, the vascular permeability returns to the previous level, or thrombosis occurs, which causes the cessation of circulation at the site of damage. The loss of plasma may cause hypovolemic shock, and the amount of fluid loss depends on the severity of the burn [12,13].

Burn wounds can be divided according to the cause, depth of the burn, surface area, and severity. Due to the cause of occurrence, burns can be divided into: chemical, electric, and thermal or radiation burns [12].

Important classification of burn wounds is based on the depth of damage, including superficial wounds (1^st^-degree), superficial partial-thickness wounds (2^nd^ degree a), deep partial-thickness wounds (2^nd^ degree b), and full-thickness burns (3^rd^ degree). Some classifications also list burns of the skin and subcutaneous tissue (4^th^ degree). Superficial burns include only epidermis and usually heal within 3–7 days without leaving scars. They are accompanied by redness and slight swelling, as well as pain that subsides after about 48–72 h. Superficial partial-thickness burns reach the papillary layer of the dermis and are characterized by soreness and the presence of blisters. They heal within 1–3 weeks (if there are no complications) and may cause long-term skin discoloration. Deep partial-thickness burns additionally involve the mesh layer of the skin. Damaged skin is red, moist, and very painful, and the epithelial process is hampered by the presence of necrosis. These types of burns heal in about 3–6 weeks, leaving scars. Full-thickness skin burns are brown, pale yellow, or red in color, and the wound surface is hard and dry, usually painless to touch. Healing leaves extensive scars [8,12,14].

The extent of a burn wound is estimated according to Wallace’s rule of nines. It is based on dividing the skin surface into regions of 9%, or a multiple of 9%, of the total body surface area (TBSA). Another method of estimating the burn area is the fives rule, which is used for children’s and infants’ burns, which divides the body surface area into regions of 5% (or a multiple). In the case of multiple burn wounds located in different places of the body, the hand rule can be used, which assumes that the area of the patient’s hand (not including the fingers) is 1% of TBSA [10,11,12,14].

The most comprehensive classification method is the burn severity assessment, using the American Burn Association’s Grading System (ABA Grading System). This system classifies wounds based on depth (degree of burn), area, burn site, and patient age (Table 1) [12].

### 2.1. Disorders of the Wound Healing Process

Healing is a complex and dynamic physiological process involving various cells, mediators, extracellular matrix components, growth factors, and proteinases. It consists of four overlapping phases: hemostasis, inflammation, proliferation, and remodeling, which are necessary for the proper course of regeneration [1,3].

Any factor that disturbs repair processes may be the cause of the pathological healing process. Factors that inhibit the healing process can be divided into: internal local (e.g., pre-existing scars), internal general (aging, diabetes, vascular diseases, poor general condition of the patient), external (infections, mechanical injuries, etc.), or mixed [3,15].

Critical factors disrupting the physiological course of chronic wound healing are the limitations of the vascularization process, causing hypoxia, resulting in prolonged and worsening inflammation and the inability of immune cells to control bacterial infection. Severe hypoxia causes the formation of necrosis, which provide a favorable environment for bacterial growth and formation of biofilm. Biofilm causes further intensification of inflammation, inhibiting the reconstruction of the extracellular matrix and tissue repair. Such a condition poses a serious threat to patients, and surgical intervention is necessary in order to debride the wound. Prolonged, increased expression of inflammatory cytokines and interleukins prevents the promotion of the healing process into the proliferation phase. Inflammation also affects the expression of metalloproteinases, which play a key role in regenerative processes, by decomposing and removing damaged extracellular matrix components from damaged tissues. In chronic wounds, excessive activity of metalloproteinases destroys growth factors, cell surface receptors, and components necessary for cell migration to the wound bed. The lack of growth factors and the presence of too many senescent cells are other problems with proper healing. Most chronic wounds heal through fibrosis, which causes formation of excessive amounts of connective tissue. The regulation of the activity of growth factors is also impaired, which causes excessive proliferation of fibroblasts, neovascularization, and excessive synthesis of collagen and fibronectin. A too strong and too long wound contraction process leads to the formation of fibrous scars [3,16,17,18,19].

### 2.2. Wound Treatment

The vast majority of wounds heal by passing through all phases of the healing properly, but, in case of some of them, this process is disturbed, causing formation of chronic wounds, requiring complicated and expensive treatment, and increasing patient mortality. Treatment methods are selected depending on the course of healing [3,17].

In the case of healthy patients, the main goal of treatment is to protect the wound against external factors (bacterial infections, mechanical injuries), accelerate wound closure by maintaining adequate humidity, and minimize scarring. In the case of patients with disorders of the physiological healing process, in addition to the aforementioned goals, it is also necessary to remove dead tissue, prevent the formation of bacterial biofilm, and modulate inflammation. Important factors that should be taken into account in wound treatment procedures to maximize patient comfort and treatment outcomes, such as: pain reduction, dressing change frequency, and treatment costs [3,20,21].

Proper preparation of the wound bed and identification of factors that adversely affect healing is extremely important. The aforementioned aspects have been included in the TIME guidelines for the management and evaluation of wounds. This integrated therapeutic strategy takes into account factors, such as: T—tissue, I—infection/inflammation, M—moisture, and E—edges. Tissue (T) includes guidelines for the assessment and debridement of the wound bed from necrotic tissue, biofilm, and adherent dressing material. Infection/inflammation (I) involves assessing the etiology and treating the infections with local or systemic antibiotics. Keeping the wound moist (M) provides instructions for assessing the intensity of exudate from this aspect of healing. Maintaining the appropriate wound edges (E) includes assessing the contraction of the wound margins and the surrounding skin. The development of an integrated TIME treatment strategy and the understanding of the molecular basis of regenerative processes have led to the development of wound healing methods and related technologies [16,20,22].

Traditional wound healing methods assume that one type of therapy is appropriate for all types of injury. As a result, the therapy is often ineffective. Standard wound care agents include topical preparations and dry dressings. The most commonly used agents are liquid preparations (solutions, suspensions, and emulsions) and semi-solid preparations (ointments and creams). The main problem with the aforementioned forms of medicine are the short duration of their activity at the site of damage, especially in the case of wounds characterized by profuse exudation [1,3,7].

A very important procedure influencing the healing process is cleaning the wound. The removal of the necrotic tissue using enzymatic substances, causing proteolytic decomposition of necrotic tissue, allows limiting surgical intervention. The first step in any treatment process is to physically cleanse the site of damage through the use of mild, aqueous solutions called lavaseptics. They are used to flush the wound in order to remove impurities, necrotic tissues, toxins, and bacteria, and their use should not cause further damage to healthy tissues. Contrary to antiseptic preparations, when using lavaseptics, it is sufficient to remove microorganisms that adhere to the wound, and it is not necessary to obtain a bactericidal effect [23,24,25].

The main antiseptics used in the treatment of wounds are iodinated polyvinylpyrrolidone (PVP-iodine), chlorhexidine, and octenidine. Octenidine dihydrochloride inhibits the growth of Gram-positive and Gram-negative bacteria and their spores, as well as acts as an antiviral, antifungal, and antiprotozoal. It has been shown that octenidine is an effective and safe antiseptic due to its high biocidal activity and high tissue tolerance. Povidone-iodine is also highly effective against Gram-positive and Gram-negative bacteria, spores, protozoa, and viruses. It works by binding to the structure of proteins and enzymes, which leads to their inactivation. There are documented cases of side effects of using this substance. A serious limitation in its use is the occurrence of the “protein error” effect, a reaction leading to a significant reduction in the antibacterial activity of this preparation in wounds characterized by high exudation. Chlorhexidine is effective against Gram-positive bacteria, protozoa, and enveloped viruses. Thus, this substance is widely used, despite reports of adverse side effects. It can inhibit tissue growth and delay healing [24,26,27].

Another widely used group of bactericides are substances containing silver ions, e.g., silver sulfadiazine, considered to be the “gold standard” in the treatment of burns [28]. Silver ions penetrate bacterial membranes and combine with DNA / RNA, which results in impaired enzymatic activity of bacteria in a very short time. Commonly used antibacterial substances in the treatment of wounds are antibiotics, such as: gentamicin, tetracycline, ciprofloxacin, vancomycin, neomycin, penicillin G, polymyxin B, amphotericin B, and mupirocin, as well as mafenide acetate [1,16,23].

The most frequently used substances promoting the regeneration of damaged tissue are growth factors, such as: EGF (epidermal growth factor), PDGF (platelet-derived growth factor), FGF-2 (fibroblast growth factor), GM-CSF (granulocyte and macrophage colony stimulating factor), and TGF-β (transforming growth factor β). In order for any of the compounds mentioned above to be effective as possible, a suitable carrier for its administration is necessary [1,7,16,23,29].

The selection of the optimal dressing is important for the regeneration of damaged tissue. Dressings can be divided into passive and active dressings. Passive dressings separate the wounds from the external environment in order to restore normal tissue function. Traditional dressings (plaster, gauze, bandage) are used to mechanically protect the wound. Modern dressings are designed not only to protect the wound but also to support ongoing repair processes and minimize complications. They are made of materials that support the healing process and can be active in the wound bed, which allows to limit the number of medications used and the procedures necessary for their application [16,30,31].

A modern dressing should have advantageous properties, such as: separation of the wound from the external environment, prevention of bacterial infections, protection against mechanical damage, acceleration of angiogenesis and reepithelialization, appropriate gas exchange between the wound and the external environment, and ensuring optimal temperature and maintenance of adequate wound moisture. Wound cleansing properties are another desirable feature of a modern dressing. Modern dressing materials should reduce therapy time and costs, adapt to the shape and movement of the body, be easy to apply and remove, be able to adjust the size to the surface of the damage, be non-toxic, and be hypoallergenic [1,23,32].

Currently, there are many modern wound treatment products on the market, mainly in the form of dressings, which increase the effectiveness of treatment, but, usually, their operation is not comprehensive and affects only individual aspects of the cascade of repair processes, which significantly reduces their effectiveness [1,23,32].

Traditionally used dressings differ in physical form and properties. They are mainly dry, so their use as primary dressings is limited. They work well as secondary dressings. One of the most commonly used material is gauze. It absorbs wound exudates well and maintains adequate humidity in the wound environment. Gauze can be used with other substances that extend the possibilities of its use, e.g., with antibiotics. Its removal can cause further injuries. This type of dressing must be replaced frequently to prevent maceration of healthy tissues. Traditional dressings are suitable for clean, dry wounds with moderate exudation. Due to the fact that such dressings do not provide a sufficiently moist wound environment, they are being replaced by modern dressings with more advanced performance [3,7,23,32].

Modern dressing materials are designed to prevent excessive drying of the wound and support healing processes. Depending on the type and origin of the wound, it is possible to select the appropriate type of dressing. Modern dressings used in clinical practice include hydrogels, hydrocolloids, films, and foams. To increase their therapeutic effectiveness, active substances can be incorporated into them [1,3,23,33].

Polyurethane films are often used as dressings. Their most important advantages include transparency, flexibility, and semi-permeability. They do not limit mobility, and they enable observation of healing progress and gas exchange, while protecting against bacterial infections. The adhesive frame surrounding the dressing ensures good adhesion and eliminates the need for an additional secondary dressing. The use of this type of dressing is limited in very exuding wounds, as it leads to an accumulation of fluid at the site of damage. The use of polyurethane foils is recommended in the case of superficial and shallow wounds characterized by low exudation [3,23].

Foams and sponges are dressings that better absorb wound exudate. Most often, they are made of synthetic polymers. This group of dressings adapts well to body movements, perfectly absorbs wound exudate, provides a moist environment, enables gas exchange, cushions in case of an injury, and thermally insulates the site of damage. They are a good dressing material for wounds with moderate or high exudate. They are usually used as a primary dressing material without the need for a secondary dressing. The high absorbency and porosity are favorable features in terms of absorption of exudate but limit the use as carriers for controlled drug release. Use of this type of dressing is limited in the case of low or no exudate because they do not ensure adequate humidity [3,23,34].

Hydrogel dressings are another type of modern dressing material. They have very high water content and are, therefore, used to treat wounds that are characterized by little or no exudation. Due to the high humidity, as well as softness and flexibility, they are easy to apply and remove from the wound without causing additional damage. These dressings do not cause irritation or tissue reactions. The biggest limitations in their use are the accumulation of exudate and the lack of gas exchange, which can lead to maceration, bacterial infections, and an unpleasant odor. This problem can be partially solved by the incorporation of an antibiotic. Anther limitation of use is dehydration, which can be limited by insulation from environment or addition of hygroscopic agents. In addition, yet another limitation in the use of hydrogel dressings is their low mechanical strength [3,23,35,36].

A kind of hydrogel dressings that play an important role among modern dressings are alginate dressings. They are biodegradable and absorb exudate well. The absorption capacity is due to the formation of a highly hydrophilic gel that reduces exudate and minimizes the risk of bacterial contamination. These dressings are suitable for treating wounds with moderate or high exudation. The alginate dressing should not be used in the treatment of dry and severe burn wounds. A secondary dressing is necessary; otherwise, the wound may become dehydrated [7,23,37].

One of the most commonly used are hydrocolloid dressings. They consist of two layers: the inner colloid layer, made of gelling agents; and the outer layer, made of elastomers and adhesives, impermeable to water. Applied to the wound, they absorb the exudate, which causes their transformation into a gel. In gel form, they adhere to the wound and are permeable to water and air, as well as provide thermal insulation and a moist environment, and they do not adhere, making them easy to remove [3,7,23].

Skin substitutes are very promising group of modern products used in the treatment of wounds. They can replace defects caused by damage. Skin substitutes can be divided into cellular and acellular. Acellular matrices are made from synthetic collagen and hyaluronic acid, or from native dermis, devoid of cellular components, preserving its architecture. Cellular skin substitutes are made of a biodegradable material (e.g., collagen, glycosaminoglycans) which is a scaffold for cells (keratinocytes and fibroblasts) that are either derived from recombinant sources or previously collected from the patient [23,38]. The morphological and mechanical parameters of this type of dressings are similar to the natural dermis. After being implanted, they gradually degrade, being replaced by a connective-tissue matrix with appropriate mechanical and structural properties. Skin substitutes provide a barrier against bacterial infections and injuries. These types of dressings can be a carrier for the controlled release of drugs [7,16,23,38].

A promising alternative to aforementioned therapies are polymeric dressings, which are also drug delivery systems. Drug delivery systems are based on different release mechanisms that can be divided into active and passive. In active systems, the release of the active substance occurs as a result of environmental or external factors. Passive drug delivery systems rely on the diffusion of the drug from the carrier or carrier degradation. Polymer controlled drug release systems can be made of degradable or non-degradable materials. This type of drug carrier is widely used due to the possibility of adjusting their action by changing the physicochemical properties of polymers [3,23,39,40].

Among the modern drug release systems used in the treatment of wounds, materials made of polymer nanofibers deserve special attention. They can be produced by various techniques, including self-assembly, phase separation, and electrospinning, but the latter is one of the most promising. Nonwoven dressing fabrics produced by the electrospinning method are characterized by a high surface to volume ratio, porosity, and the ability to absorb exudate, without its accumulation directly at the site of damage, ensuring good gas exchange, and protecting the wound against infections and dehydration. Polymer nonwovens can be made of natural polymers, but interesting materials used in their production are biodegradable polyesters and polyester carbonates. The incorporation of the active substance into the polymer fibers allows for the limitation of the local supply of the drug substance and the controlled release of the drug at the site of damage. The fabrication of nonwovens from degradable materials allows reducing the need of dressing replacement. Due to this, nonwovens are characterized by increased therapeutic effectiveness and increase in the patient’s comfort of life [1,3,6,7,32,41].

Other important wound treatment methods include hyperbaric oxygen therapy and negative pressure wound therapy (NPWT). Maintaining a negative pressure at the site of injury promotes wound closure. The wound is treated with an airtight dressing connected to a vacuum-generating apparatus, which ensures a stable environment. Low pressure limits shear forces that can damage newly-built tissue. The pressure gradient creates a mechanical stress which induces an effect known as stretching of the cells. Cell proliferation and tissue maturation are accelerated, resulting in faster vascularization, collagen deposition, and granulation formation. Negative pressure wound therapy ensures a moist wound environment, while allowing exudate drainage. Hyperbaric oxygen therapy is based on the use of pure oxygen at higher than atmospheric pressure. The patient is placed in a pressure chamber. Hyperoxia leads to vasoconstriction, promotes angiogenesis and multiplication of lymphocytes, reduces the action of toxins, and works synergistically with antibiotic therapy [32,38].

Treating complex wounds remains a challenge as current therapeutic strategies do not provide comprehensive solutions. The development of controlled drug release carriers has allowed a new look at the issues related to wound therapy; these carriers enable sustained drug release and improve tissue response to treatment. Moreover, some of them are characterized by a synergistic effect, as they not only provide the active substance but also imitate natural tissue and create optimal conditions for healing processes. Despite a number of advantages, they also have some limitations in use, due to the complicated production procedures, and it is complicated to assess their biocompatibility, toxicity, and therapeutic effectiveness [3,39,40].

## 3. Propolis

Apitherapy is based on the use of biological properties of bee products in the prevention and treatment of many human diseases. Many of the techniques used to heal wounds in modern medicine are not very different from traditional practices [42].

One of the natural substances of bee origin, used in medicine for centuries, which can be incorporated into polymer fibers in order to obtain biodegradable nonwovens for dressings, is propolis [43]. The name of this substance, derived from the Greek parts “*pro*”, meaning “in front of”, and “*polis*, meaning “community”or “city”, indicates its protective functions [44]. It is a sticky, plant substance formed from resins that honey bees collect from plants and add secretions from the pharyngeal and mandibular glands. Bees use propolis to seal cracks, smooth the walls, maintain a constant temperature and humidity in the hive, and to protect the larvae, honey, and combs against microbial contamination [44,45]. Numerous studies show that propolis owes its activity to the synergistic action of its numerous components [45]. Propolis has a very wide spectrum of activity; it is used as an antibacterial, anti-inflammatory, antiviral, antiprotozoal, antifungal, antiseptic, analgesic, antitumor, antioxidant, antimutagenic, and antihepatotoxic agent [44]. Research has also shown the beneficial effects of propolis on wound regeneration and the prevention of scarring [46,47,48,49,50].

### 3.1. Composition

About 300 components of propolis have been identified so far [51]. The content of individual components in this material is as follows: plant resins, 50%; beeswax, 30%; pollen, 5%; essential and aromatic oils, 10%; and other organic compounds [44]. Its composition depends on the geographic region, as well as the harvest time. In European countries, propolis comes mainly from black poplar buds, which makes it classified as poplar-type propolis [52]. Among the marked compounds, the following groups of substances can be distinguished: phenolic acids, flavonoids, terpenes, lipid-wax substances, beeswax, bioelements, vitamins, proteins, sugars, and amino acids. Both in terms of quantity and quality, the most numerous group of compounds in propolis are polyphenols [53]. Another important group of compounds in propolis are flavonoids, which are essential in terms of its action [54]. The most important flavonoid compounds in Polish propolis include: quercetin, apigenin, tectochrysin, pinocembrin, chrysin, genquvanin, galangin, kaempferol, and 5-hydroxy 4′,7-dimethoxyflavone [53,55].

### 3.2. Standarization and Quality Control

An important challenge in the application of the discussed apitherapeutic product on a mass scale is the establishment of standardization and quality control procedures, which scientists have been working on for years [56,57,58]. The geographical region where bees collect the ingredients to produce propolis is an important factor influencing its composition and, thus, the spectrum of its activity. In the 1990s, it was proposed to characterize dry propolis by estimating the total content of phenolic substances, flavonoids, waxes, ash, volatile substances, and dry residue, as well as to characterize propolis tinctures by estimating the total content of phenolic substances, flavonoids, waxes, specific gravity, and ethanol [59]. Currently, the chromatographic methods are the methods of choice for the analysis of the most important propolis components, particularly high performance liquid chromatography combined with a photodiode array (HPLC-PDA) or mass spectrometry (HPLC-MS) [60,61]. Additionally, techniques, such as capillary electrophoresis (CE), thin layer chromatography (TLC), gas chromatography (GC), and nuclear magnetic resonance (NMR), are also being used for the analysis of propolis [61]. The potential mass use of apitherapeutic agents would also require controlling the authenticity of a final product. The solution to this problem may be the methods of principal component analysis (PCA) and fingerprint analysis, which can be used as a reference in the verification of the authenticity and quality control of a given product [62]. The latest research proves that, apart from determining the total content of phenolic compounds and flavonoids, it is also important to test the phenolic-associated in vitro activity of propolis sample [63]. The appropriate selection of quality control methods for propolis-based products guarantees its appropriate composition and, thus, a specific spectrum of activity of obtained product.

### 3.3. Activity

**Figure 1:**
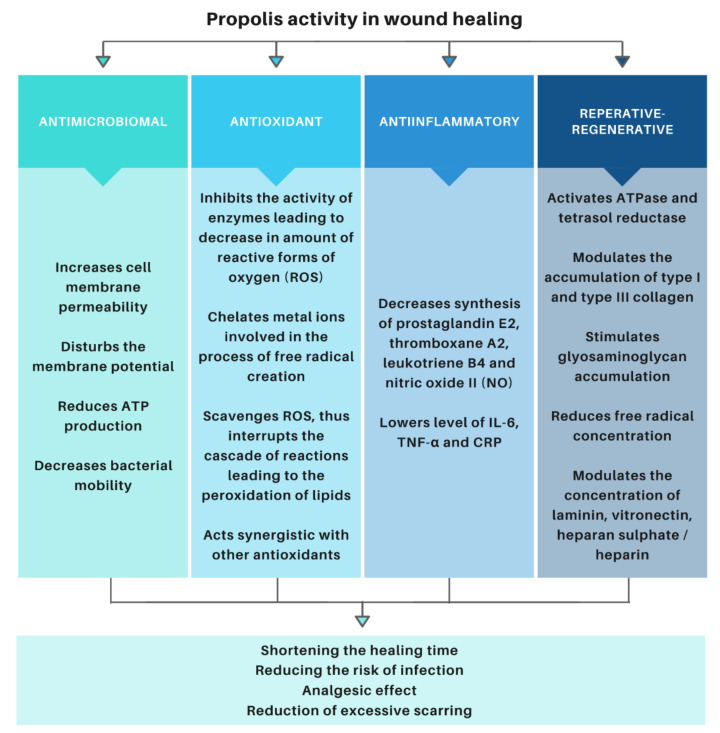
Mechanism of propolis action in wound healing.

Propolis has a number of activities that have a beneficial effect on wound healing (Figure 1).

The phenolic compounds contained in propolis are natural, exogenous antioxidants. Their mechanism of action is based on inhibiting the activity of enzymes, thus inhibiting the formation of reactive oxygen species (ROS), chelating metal ions involved in the formation of free radicals, capturing reactive oxygen species, and breaking the cascade of reactions leading to lipid peroxidation, as well as synergistic action with other antioxidants [53,64,65]. Antioxidant properties of propolis have been proven in numerous studies using methods, such as DPPH-radical scavenging activity, ABTS^+^-radical scavenging activity, Ferric Reducing/Antioxidant Power assay (FRAP), and ORAC assay [28,56,66,67,68,69,70,71,72].

The anti-inflammatory activity of propolis has been demonstrated in both acute and chronic inflammation. It is a consequence of the aforementioned aspects of the antioxidant activity. The anti-inflammatory effect is related to the inhibition of the synthesis of compounds involved in inflammatory reactions [73,74,75]. It has been proven that the anti-inflammatory effect of propolis is the same as in the case of non-steroidal anti-inflammatory drugs (NSAIDs), but it does not cause such side effects [53,76,77].

Antibacterial properties of propolis are the subject of numerous scientific studies [50,78,79,80]. Antibacterial activity results from the synergistic action of many of its compounds [50]. Propolis acts by damaging the structures and disrupting the function of the cell wall and cytoplasmic membrane of bacteria. It also inactivates enzyme proteins located in the cytoplasmic membrane and disrupts the transport of nutrients and the synthesis of cellular components. Propolis also induces a reduction in the membrane potential by changing the permeability of the cell membrane, which leads to a disruption of the proton pump and the reduction of ATP (adenosine-5′-triphosphate). These processes lead to reduced mobility or complete immobilization of ciliated bacteria. All of the aforementioned aspects of the action of this substance lead to the lysis and death of bacteria [53,81]. The studies showed greater antibacterial activity against Gram-positive than Gram-negative bacteria. *Staphylococcus aureus* strains are most often used to assess the antibacterial activity of propolis, due to their strong activity against this strain. Propolis also inhibits strains: *Mycobacterium tuberculosis*, *Mycobacterium avium*, *Staphylocuccus epidermis*, *Staphylococcus pyogenes*, and *Klebsiella pneumoniae* [82,83]. It has been shown to be bactericidal against strains of *Bacillus* spp., *Enetrococcus faecalis*, and fungicidal against *Candida albicans* [84]. The antibacterial effect of propolis is extremely important in the treatment of wounds, as much as 75% of deaths due to burns are caused directly by wound infection [85].

The comprehensive action of propolis components has a positive effect on the treatment of wounds [76,86,87,88,89,90,91,92]. The results of the research confirmed the therapeutic efficacy of this bee-derived product in comparison to silver sulfadiazine in the treatment of thermal injuries through quantitative and qualitative assessment of the accumulation of type I and III collagen in the damaged tissue matrix. The studies showed that phenolic components modulate the accumulation of type I and III collagen at the site of thermal damage. By stimulating the reconstruction of the collagen matrix at the burn site, the use of propolis promotes re-epithelialization and creates a favorable biochemical environment supporting wound healing, as well as minimizes keloid formation and excessive scarring [47]. Propolis influences the reconstruction of tissue by modulating the granulation process. It is related to the stimulation of glycosaminoglycans (GAGs), such as: dermatan sulphate (DS), chondroitin sulphate (CS), and hyaluronic acid (HA) [49].

Biochemical analyses have shown that the use of propolis causes an increase in the concentration of heparan sulphate / heparin and non-collagen glycoproteins, such as laminin (LN) and vitronectin (VN), in the initial phase of the experiment, followed by a reduction in the number of tested molecules, which contributed to more effective control healing at the cellular level [48]. Propolis also has a beneficial effect on the metabolism of fibronectin by inhibiting the biosynthesis of native fibronectin and reducing its degradation in damaged tissue [93]. It has been shown that, in burn wounds treated with propolis, the concentration of free radicals is lower than in wounds treated with silver salt of sulfadiazine [94].

The therapeutic effectiveness of propolis extracts applied in various media against burn wounds was also confirmed by the histopathological assessment of tissue specimens. The effectiveness of this apitherapeutic agent in the treatment and prevention of pressure ulcers has also been confirmed [80,95]. Modulation of the amount of extracellular matrix components at various stages of regeneration of damaged tissue significantly accelerates the healing process and reduces scarring.

Propolis is active against mast cells, which play an important role in all stages of healing. It has been proven that it causes a statistically significant reduction in the number of mast cells, both at the edges and in the central part of the postoperative wound in the acute phase of infection, compared to dexamethasone [96]. Limiting the number of mast cells, which, in excessive density, impedes healing and leads to the formation of keloids, reduces inflammation and scarring [50].

The therapeutic efficacy of propolis has also been proven in clinical trials. In an 18-week randomized controlled study, glucose metabolism and antioxidant function of Brazilian green propolis in patients with type 2 diabetes mellitus (T2DM) was evaluated. It was concluded that examined propolis is effective in improving antioxidant functions in T2DM patients [97]. In a double-blind, placebo-controlled clinical trial, the effects of the oral administration of propolis solution on the oxidative status modulation of lipids was evaluated. The results obtained show a positive influence of propolis on oxidative status and improvement of HDL-c [98]. Randomized placebo-controlled study assessed the effect of propolis as an adjuvant in the healing of human diabetic foot ulcers. The study showed that propolis improve and promote wound healing [99]. A randomized controlled study on effect of propolis topical application on wound healing after tonsillectomy showed beneficial effects in reducing postoperative pain, preventing hemorrhage, and accelerating wound healing [100]. It has been also shown that propolis can become alterative treatment option for chronic periodontitis during supportive periodontal therapy [86].

Due to the proven beneficial effect of the apitherapeutic agent on the various aspects of wound treatment, new methods of administering this substance are constantly being sought. Except the commonly used ointments, foams, or creams, an increasing number of research studies are focusing on the drug delivery systems (DDS) of this apitherapeutic, such as: nanoparticles, membranes, organogels, and nonwovens, which will allow overcoming the limitations associated with the forms of this substance used so far [79,101,102].

## 4. Biodegradable Polyesters

Today, biodegradable polymers are used as therapeutic temporary implants, such as surgical sutures, internal bone fixation products, drug delivery systems, and scaffolds for use in tissue engineering. Biodegradable polymers can be divided into two main categories: (1) natural polymers (biopolymers), produced by plants, animals, and microorganisms, which include: cellulose, starch, chitin, polyhydroxyalkanoates; and (2) synthetic polymers, for example: polylactide (PLA), poly-(Ɛ-caprolactone) (PCL), and polyglycolide (PGA). Synthetic polymers have a number of advantages: they can be produced industrially on a large scale, and their chemical properties can be modified depending on their potential application [103,104].

Biomaterial is defined as a material designed to work with biological systems to research, heal, support, or replace any tissue, organ, or function in the body. The basic condition that a biomaterial must fulfill is a biocompatibility. The response to an implant depends on a number of parameters, ranging from material properties to the shape and structure of its final form. The properties of the degradable material change over time as the degradation products differ in properties from the initial material. The most important properties of the biomaterial include: no prolonged inflammatory reaction after implantation, adequate degradation time and mechanical properties, and degradation to non-toxic products that can be metabolized and removed from the body. The technological possibilities of transforming the material into a product suitable for a given application are also important [104].

The use of synthetic polymers is very wide due to the simplicity and low processing costs. Biodegradable synthetic polymers are the subject of numerous scientific research studies in the field of medicine due to the lack of the need to remove implants made of them from the patient’s body. Biodegradable polyesters are the most used materials in tissue engineering due to their high tissue compatibility, which limits the body’s inflammatory response [16,105].

Delivery of drugs through polymer carriers allows the properties of the controlled release system to be adjusted by changing the physicochemical properties of the polymer used. Parameters affecting the kinetics of drug release are: chemical structure, the length of the polymer chains, glass transition temperature (Tg), polymer chain microstructure, crystallinity, hydrophilicity, and polymer degradation rate. Molecular weight affects the viscosity of the polymer melt or solutions, degradation rate, and mechanical properties of the material. Lower molecular weight polymers degrade in shorter period of time; thus, release of the active compound occur faster. Glass transition temperature of a polymer (Tg) is the temperature at which the transition of the amorphous regions from a glass to a plastic state occurs. At temperatures below the Tg, the amorphous regions are glassy, and diffusion through them is limited, slowing the release of the active ingredient. An important parameter of the polymer is its crystallinity, since diffusion takes place in amorphous regions [3,39,106].

Another important parameter of the material is its hydrophilicity. Hydrophobic materials tend to erode on the surface, while hydrophilic materials are more prone to swelling, which facilitates mass degradation, but the surface to volume ratio of the final form of the material also influences the type of degradation mechanism. Surface erodible materials are characterized by a higher rate of degradation and weight loss at the phase boundary between water and polymer material, than by the rate of water diffusion into the material; therefore, degradation takes place only at the surface. In the case of bulk erosion, this phenomenon is the opposite; the diffusion of water into the material is faster than the degradation rate, which means that it occurs not only on the surface but in the entire volume of the material. Moreover, the mass degradation of biodegradable polyesters results in acidification with decomposition products and autocatalysis of the hydrolysis of ester bonds; therefore, there is a sudden acceleration of degradation. Surface eroding materials are excellent for sustained drug release because they have near-zero-order release kinetics that can be easily adjusted. Bulk degrading materials maintain adequate diffusion, making them suitable for applications that require permeable material, such as tissue engineering [3,39,106,107].

Numerous studies have proven that the microstructure of the copolymer affects its properties and, thus, the parameters of the obtained product. Chemical structure and polymer chain microstructure has been modified by changing the amount and type of comonomers used for the synthesis and the adjustment of its conditions, such as temperature and duration of the reaction, as well as the type and amount of initiator [103,108,109,110,111,112,113]. It has been shown that the use of the low-toxic Zr(acac)_4_ initiator leads to a more segmented copolymer structure than in the case of the Sn(oct)_2_, conventionally used in PLGA copolymerization [109]. Polymer chains with a higher degree of randomization undergo faster hydrolytic degradation [113]. In the studies conducted by Jelonek et al., a detailed analysis of changes in the microstructure of the poly(lactide-co-trimethylene carbonate) PLATMC chain during degradation and their impact on the release of cyclosporin A and rapamycin were analyzed. It was shown that the microstructure of the copolymer chain determined the influence of the drug content on the polymer degradation process. It has also been concluded that highly randomized copolymers release the active substance evenly, and a decrease in the randomization rate may cause fluctuations in drug release [114]. The research carried out by Orchel et al. showed that the poly(lactide-co-glycolide) (PLGA) 85:15 copolymer, which has the most segmented structure among the studied materials, turned out to be the best material for chondrocyte culture [115]. The experiment carried out by Jawroska et al. showed that the use of polymers with an appropriate microstructure to cover medical implants enables the controlled release of ciprofloxacin from their surface, thanks to which obtained product has antibacterial properties [116]. Moreover, the studies carried by Jelonek et al. have proven differences in the sirolimus release profiles from the same composition polymer coatings applied to polymer scaffolds of different composition [117]. The above examples have proven that the microstructure of the polymer is one of the key parameters influencing the degradation of the material and the release of the active substance.

The materials most commonly used for production of drug-releasing dressing materials are biodegradable polyesters, such as PLA, PGA, PCL, and their copolymers, such as PLGA and poly(lactide-co-caprolactone) (PLCL) [118]. They have aliphatic ester bond, which causes that, mainly, polyesters with short aliphatic chains are used for biomedical applications. The stability of this type of bond also causes them to degrade in mass, although they are slightly hydrophobic [107]. Due to the simplicity of synthesis and wide commercial availability, aliphatic polyesters are among the best-studied biodegradable biomaterials. The uniqueness of this class of materials lies in its great variety and versatility. Polyesters can be obtained by ring-opening polymerization, as well as by polycondensation [107,118].

Polyglycolide is an aliphatic polyester with a simple structure, characterized by a glass transition temperature about 35–40 °C, a melting point about 200 °C, and high crystallinity, which makes it poorly soluble in aqueous solutions. It is one of the first polyesters tested for biomedical applications. Few studies have been done on PGA-controlled drug release systems due to their poor solubility in common solvents and very fast degradation (approximately 4 weeks). In studies on this material, scientists are focusing on its use in combination with other degradable polymers as a short-term tissue engineering scaffold. PGA has so far been used as a filling material for cartilage, bones, teeth, and tendons. It is also used as a degraded suture. However, there are several significant problems with use of poly(glycolic acid): rapid degradation results in a loss of mechanical properties and local accumulation of glycolic acid, which can cause a strong inflammatory response, even though it is metabolized by cells in the citric acid cycle [3,16,103,107].

Polylactide is structurally similar to PGA (it differs in having a methyl group on the α carbon); however, it differs in physical and mechanical properties. It is more hydrophobic, and it hydrolyzes more slowly. PLA has chiral molecules in its structure, so it occur in forms of: poly(L-lactide) (PLLA), poly(D-lactide) (PDLA), racemic mixture—poly(D,L-lactide) (PDLLA), and meso form. This polymer is mechanically stronger than PGA. Depending on the share of the aforementioned forms, it differs in Tg, degree of crystallization, and mechanical properties. Only PLLA and PDLLA are promising for biomedical applications and have been extensively studied. PLLA has a glass transition temperature of about 60–65 °C and a melting point of about 175 °C, while PDLLA has a Tg of about 55–60 °C and has a slightly lower mechanical strength than PLLA. As the degradation time for high molecular weight PLLA is even longer than 5 years, various techniques have been developed for modifying, copolymerizing, and blending PLLA with other degradable materials. Modified PLLA is used as a controlled drug release system due to the ability to control its degradation time and, thus, to control the kinetics of the release of the active substance. Moreover, PLLA has also found application as a scaffolding material for the regeneration of bones, tendons, cartilage, blood vessels, and nerve cells. The degradation time for PDLLA is about a year, so it is also rarely used as a standalone drug release system. It is used in tissue engineering or combined with other degradable polymers. Polymers that are used to accelerate PLA degradation are: PLGA, polyethylene glycol (PEG), chitosan, and collagen [3,16,105,107].

Poly(ε-caprolactone) has a very low glass transition temperature of about −60 °C. It is soluble in organic solvents. Due to the very long degradation time in vivo and the high permeability, it is used as a carrier for the long-term drug delivery. Its degradation time is approximately 2–3 years. To accelerate this process, it is copolymerized or blended with other polyesters. Although the use of this material in drug-controlled release systems is limited, it is widely used in tissue engineering due to its mechanical properties. This material has a very high tensile elongation value and low tensile strength, which makes it very flexible. Its high plasticity can be modulated by mixing with PLA, PGA, and PLGA to increase its strength. Tissue engineering scaffolds from PCL are manufactured using microsphere adhesion, porogen leaching, or electrospinning. Poly(ε-caprolactone) and its composites are used in tissue engineering to regenerate bones, cartilages, ligaments, vessels, skin, and nerves [16,103,105,107].

PLGA copolymer is obtained by copolymerization of lactide and glycolide. It is currently the most widely researched degradable polymer for medical applications. Release of the active substance from poly(lactide-co-glycolide) initially proceeds with a burst effect and is then characterized by zero order kinetics. The initial burst effect is caused by water diffusion into the material, which causes elution of the active substance. Release profiles differ depending on many factors, particularly hydrophilicity of the drug, molecular weight of the polymer, and the mutual monomer ratio [3,119,120,121]. PLGA with a lactide content from 25% to 75% is a polymer with an amorphous structure, which causes its hydrolytic instability compared to the more stable PLA homopolymer. Degradation times of copolymers with mutual ratios of lactidyl subunits to glycolidyl subunits are, respectively: about 1–2 months for PLGA 50:50, 4–5 months for PLGA 75:25, and 5–6 months for PLGA 85:15. PLGA is widely used in the fabrication of resorbable surgical sutures, tissue engineering scaffolds, and drug-controlled release systems. The selection of the copolymer composition allows for its optimization in terms of the intended use. As a drug carrier, this material is used to deliver chemotherapeutic agents, antibiotics, analgesics and anti-inflammatory drugs, vaccines, proteins, and siRNA. The most common products made of this copolymer are microcapsules, nanospheres, and nonwovens. This material is also perfect for tissue engineering due to its excellent adhesive and proliferative properties. Tissue scaffolds made of PLGA are produced using techniques, such as gas foaming, porogen leaching, microsphere sintering, 3D printing, and electrospinning. So far, they have been used in the cultivation of cells of bone, cartilage, skin, tendons, nervous tissue, and liver cells [104,107,121,122].

When it is necessary to use a biodegradable material with high flexibility, it is possible to use a copolymer or blend of PLA with a polymer with a low glass transition temperature, such as poly(ε-caprolactone), in which elongation at break exceeds 700%. Due to the low miscibility of these polymers, the formation of their blends results in phase separation. An effective solution is their copolymerization, which allows to control the mechanical properties of the material, degradation rate, and drug release profile [123,124].

Another elastomer used for biomedical applications is poly(trimethylene carbonate) (PTMC). It is characterized by a glass transition temperature of about −17 °C, high flexibility, and low mechanical strength. Due to the presence of the carbonyl bond, its hydrolytic degradation is slow. It has been reported that it undergoes enzymatic degradation in vivo, which causes surface erosion. In order to increase its applicability, it is often copolymerized with PLA, PCL, polyether, and poly(L-glutamic acid), as well as both with PLA and PGA. PTMC is used to produce discs, microparticles, and gels. Its copolymers can be used to make products which must have much better mechanical properties, e.g., surgical sutures. Among the substances delivered by this polymer and its copolymers, there are angiogenic factors, antibiotics, and chemotherapeutic agents [104,107].

Recently, a terpolymer consisting of lactide, glycolide, and trimethylene carbonate, with a mutual ratio of comonomers amounting to approximately 75% lactidyl subunits, 10% glycolidyl subunits, and 15% trimethylene carbonate subunits, has been increasingly used. A unique feature of this material is that it returns to its original shape in the temperature of the human body as a result of shape memory effect. This material can be used as a self-expanding stent or a carrier for drug delivery, and it has been widely characterized in terms of synthesis, polymer chain microstructure, thermal properties, and degradation in vivo [124,125,126,127,128].

Today, many biodegradable, biocompatible polymers are used as biomaterials. Combining them enables obtaining material with the desired properties for very specific applications. Development of processing techniques enables the manufacturing of products with a very complex architecture that can imitate biological structures. The advantage of the biodegradable polymers over other materials lies in the possibility of designing or modifying them in order to obtain the appropriate degradation time, active substance release profile, and mechanical properties. The continuous development of biomaterials and numerous studies indicate the possibility of their application in the treatment of wounds and the achievement of promising results in this field [104,107].

## 5. Electrospinning

The use of fibrous materials is constantly expanding in areas, such as controlled drug release, tissue engineering, and regenerative medicine, as well as in many others [88]. Nonwovens are promising materials for wound healing dressings. Polymer fibers can be obtained using various methods, such as phase separation, template synthesis, molecular self-assembly, or electrospinning. Among the methods mentioned above, the electrospinning method is a unique technique because of the simplicity of implementation. It is a simple and versatile technique that allows the production of nano- and microfibers that provide a favorable environment for cell growth due to its similarity to the native extracellular matrix [84]. Polymer nonwovens obtained by the electrospinning method are becoming more and more popular due to favorable properties, such as: the possibility of obtaining very long fibers, a high surface-to-volume ratio (large surface area), high porosity, excellent mechanical properties, and high biocompatibility. This method enables the processing of both natural and synthetic polymers. It is possible to obtain fibers with a diameter ranging from a few nanometers to several micrometers. Among all the methods of forming nanofibers, it is this technique that allows the most effective production of materials with a uniform structure [129,130].

### 5.1. Principle of the Method

Electrospinning is the production of fibers from polymer melt or solutions in an electric field. It is an electrohydrodynamic process in which a drop of liquid is exposed to an electric field, which causes drop deformation and fiber formation. The basic equipment for electrospinning consists of a high voltage source (power supply), an infusion pump, a spinning nozzle, which is usually a blunt-ended metal needle, and a conductive collector [131].

The polymer is pumped with a constant flow rate through a spinning nozzle. Due to surface tension, a drop is formed. Power supply generating the electric potential is connected to the nozzle, so that the surface of the droplet is electrostatically charged. Two electric forces act on the droplet which causes its deformation into a cone: mutual electrostatic repulsion of charges on the surface and the Coulomb interaction exerted by an external electric field. In contrast, the surface tension and forces resulting from the viscoelastic properties of the liquids cause the drop to maintain a spherical shape. When equilibrium is reached between these forces, the liquid at the end of the capillary takes the shape of a Taylor cone. When the critical value of the applied electric potential is exceeded, a jet of polymer solution is ejected from the end of the cone [131,132].

The stream of polymer solution ejected from the cone is directed towards a grounded or oppositely charged collector as it tends to close the electrical circuit. Initially, its course is linear, but, as it moves away from the cone, it becomes disturbed, becoming unstable, and then completely chaotic. At the same time, the streams are elongated, which leads to their stretching to smaller diameters due to the potential difference. This facilitates the evaporation of the solvent (in the case of electrospinning from the solution) and lowering the temperature (in the case of melt electrospinning), which leads to solidification of the fibers and their deposition on the collector [131,132].

### 5.2. Biomedical Applications of Eletrospinning Process

The interest in fibrous materials produced by the electrospinning method is constantly increasing because they perfectly reflect the surface and morphology of the extracellular matrix. The cellular response to the biomaterial is better when the morphology of the used cellular scaffold matches the native tissue well. Due to the possibility of choosing different materials and, thus, the possibility of giving the product specific mechanical and biomimetic properties depending on the intended use, nonwovens are finding more and more biological applications. Today, researchers are focused on fabricating scaffolds for tissue engineering, dressings, drug delivery systems, and enzyme immobilization [118,133].

Cell culture scaffolds made of synthetic polymers are becoming more and more popular. The ideal material is poly(lactide-co-glycolide), which is biodegradable, easy to spin, and has the ability to change its properties. It has also been shown that poly (ε-caprolactone) (PCL) nanofibers are excellent for bone regeneration [118,133]. Numerous studies have shown the usefulness of nonwovens obtained by the electrospinning method as carriers for the controlled release of active substances. Their high efficiency as scaffolding materials in tissue engineering and as dressing materials in regenerative medicine has also been demonstrated. The greatest advantages of this type of materials are the ability to control their parameters through modifications at various stages of their production from the selection of the material, through the selection of the active substance, to the optimization of process parameters, enabling the obtaining of nonwovens with properties appropriate for a given application [118,134].

One of the most important challenges faced by modern drug carriers is to deliver the active ingredient in the most physiological manner. Reducing the size of a drug form and producing it from an appropriate material increases the capacity of absorption of the drug at the site of action. The possibilities of drug delivery using this type of carriers are enormous. However, the process and carrier parameters should be adapted to the requirements of the application. The drug can be incorporated into the fibers, as well as coated on their surface. It is also possible to create layered nonwovens, interlacings, coaxial fibers, and a number of other modifications that change the drug release profile. Nonwovens are also characterized by higher therapeutic efficacy and lower toxicity [118,124,131,133].

Electrospun nonwovens containing propolis are the subject of numerous studies (Table 2).

Our own research in the field conducted so far also indicates the possibility of using biodegradable nonwovens with propolis for the treatment of wounds. Electron paramagnetic resonance (EPR) spectroscopic examination of different types of paramagnetic centers in the blood during healing of burn wounds revealed that PLGA nonwoven dressings strongly influence the oxidative-antioxidative balance during the burn wound healing process [146]. A favorable effect of innovative biodegradable apitherapeutic dressings on burn regeneration has been proven, as evidenced by changes of blood paramagnetic centers and free radicals, suggesting a pluripotent multifaceted influence of propolis contained in nonwovens on oxidative balance changes [147]. It has been also shown that release of the propolis is related to degradation of polymer carrier; the sooner the degradation occurs, the faster the active compound will be released. The difference in drug release kinetics between samples at the early stages of incubation is very important because it allows choosing the appropriate release profile to saturate the damaged tissue. The beneficial effect of the discussed nonwovens on the treatment of burn wounds in vivo has also been proven [145].

## 6. Conclusions

The analyzed research results indicate that development of completely biocompatible and biodegradable polymer dressings that release a substance with great therapeutic potential, propolis, would significantly contribute increasing the effectiveness of wound treatment, as well as improving the patient’s quality of life. This is supported by a number of beneficial properties of the dressing:

- The use of well-tested biodegradable polyesters makes the dressing biodegradable and biocompatible. The knowledge of the degradation processes and the release profiles of the drug substance allows for the selection of the appropriate material composition. The production of a dressing from a material that degrades and releases the active substance during wound healing enables its selection to avoid the necessity of dressing changes and supply of the active substance.
- Propolis is an apitherapeutic agent with documented antibacterial, antiviral, antifungal, antitumor, anti-inflammatory, analgesic, and repair and regenerative properties. The controlled release of propolis throughout the treatment period accelerates healing, reduces the risk of infection, and improves the cosmetic effect by reducing scar formation.
- Electrospinning is a method of producing nonwovens that mechanically protect the wound and mimics ECM, while allowing gas exchange within the wound. Moreover, it allows for the adaptation of the release profile and the time of complete degradation by modifying the production parameters.

Research on the aforementioned dressings is in line with contemporary trends in developing more and more effective methods of wound treatment.

## Figures and Tables

**Table 1:** Classification of burn wounds based on the American Burn Association’s Grading System.

Type of Wound	Description
Minor	Partial-thickness < 10% TBSA of children ^1^ and elderly
	Partial-thickness < 15% TBSA of adults
	Full-thickness < 2% TBSA
Moderate	Partial-thickness 10–20% TBSA of children and elderly
	Partial-thickness 15–25% TBSA of adults
	Full-thickness 2–10% TBSA
Major	Partial-thickness > 20% TBSA of children and elderly
	Partial-thickness > 25% TBSA of adults
	Full-thickness > 10% TBSA
	Burns in critical areas ^2^
	Complicated burns ^3^

^1^ Children < 10 years old, adult 10–40 years old, elderly > 40 years old. ^2^ Critical areas: face, perineum, hands, and feet. ^3^ Complications: high-voltage electrical burns, inhalation injury, associated major trauma and comorbidities.

**Table 2:** Research on electrospun nonwovens containing propolis taking into account their potential applications and the scope of research.

Material	Propolis Content	Application	Scope of Research	Ref.
Polyvinylpyrrolidone (PVP)	5% (*w*/*v*)	Mouth-dissolving dosage form and an anticariogenic agent in the oral cavity	Fiber morphology (SEM) Antibacterial activity Contact-angle Disintegration/Dissolving Time	[135]
Cellulose acetate (CA), Polycaprolactone (PCL)/Cellulose acetate (CA)		Wound healing	Fiber morphology (SEM) Water absorption Contact-angle ATR-FTIR analysis Antioxidant assay Antibacterial activity	[136]
Polyamide-6 (PA-6)	20%, 30%, 40%, 50% (*w*/*w*)	Medicine and food industry	Thermal analysis (TGA, DSC) FTIR analysis Fiber morphology (FESEM) XRD analysis Drug release Antioxidant assay	[137]
Honey/polyvinyl alcohol (PVA) / chitosan (HPCS) 30:7:3.5	10% (*w*/*w*)	Wound dressing	FTIR analysis Fiber morphology (FESEM) Antibacterial activity In vivo wound-healing Histological examination Cell viability Cell proliferation	[79]
Polyvinyl alcohol (PVA)	5%, 10%, 20%, 40%, 60% (*w*/*w*)	Wound dressing	Fiber morphology (SEM) FTIR analysis XRD analysis Loading efficiency Drug release Water absorption Weight loss Antibacterial activity	[138]
Zein	5%, 10%, 15%, 20%, 25%, 30% 35%, 40% (*w*/*w*)	Wound dressing	Fiber morphology (SEM) Antibacterial activity FTIR analysis	[139]
Polyurethane (PU)	5%, 10%, 30% (*w*/*w*)	Wound dressing, tissue engineering	Fiber morphology (FESEM) FTIR analysis Mechanical properties Contact-angle Antibacterial activity Cell viability	[101]
Polyvinyl alcohol (PVA)	10%, 30%, 50% (*w*/*w*)	Controlled delivery system	Fiber morphology (SEM) Drug release	[140]
Polyvinyl alcohol (PVA) / Gelatin (Gel) 13%:0.5% (*w*/*w*)	3%, 5% (*w*/*w*)	Corneal patches	Fiber morphology (SEM) Thermal analysis (DSC) Antibacterial activity Mechanical properties Drug release Cell viability Contact-angle	[141]
Polyurethane (PU) / Hyaluronic acid (HA) 1:1	0.5%, 1%, 2% (*w*/*w*)	Wound dressing	FTIR analysis Thermal analysis (TGA) Fiber morphology (SEM) Mechanical properties Contact-angle Water absorption Drug release Cell viability Cell morphology In vivo wound-healing Histological examination	[142]
Poly(ethylene oxide) (PEO) / hyaluronic acid (HA)	7% (*w*/*w*)	Wound dressing	FTIR analysis Fiber morphology (SEM) Water-vapor transmission rate Antioxidant assay Cell viability Antibacterial activity	[143]
Poly(3-hydroxybutyrate-co-3-hydroxyhexanoate) (PHBH)	1%, 5%, 7% (*v*/*v*)	Wound dressing	Fiber morphology (SEM) FTIR analysis XRD analysis Mechanical properties Drug release Antibacterial activity	[144]
Polylactide (PLA)	10%, 20% (*w*/*w*)	Wound dressing	Fiber morphology (SEM) FTIR analysis Contact-angle Drug release Antimicrobial activity Cell viability	[43]
Poly(lactide-co-glycolide) (PLGA)	5%, 10% (*w*/*w*)	Wound dressing	Fiber morphology (SEM) Water absorption Weight loss Changes in polymer composition (NMR) Drug release In vivo wound-healing	[145]

SEM = Scanning Electron Microscopy, ATR-FTIR = Attenuated Total Reflectance-Fourier Transform Infrared Spectroscopy; TGA = Thermal Gravimetric Analysis; DSC = Differential Scanning Calorimetry, FTIR = Fourier Transform Infrared Spectroscopy; FESEM = Field-Emission Scanning Electron Microscopy; XRD = X-Ray Diffraction; NMR = Nuclear Magnetic Resonance.

## Data Availability

Not applicable.
